# Eating habits, nutritional status, and lifestyle correlates of body composition in Chinese university students: a cross-sectional study

**DOI:** 10.3389/fpubh.2025.1668307

**Published:** 2025-11-12

**Authors:** Ling Liu, Wan Gong, Jun Li, Wei Zhou, Li Lin

**Affiliations:** 1 School of Clinical Medicine, Sichuan College of Traditional Chinese Medicine, Mianyang, Sichuan, China; 2 Institute for Nutrition and Food Safety, Sichuan Center for Disease Control and Prevention, Chengdu, Sichuan, China

**Keywords:** dietary habits, hidden obesity, low protein mass, fruit intake, nutrition intervention

## Abstract

**Background:**

Unhealthy eating habits and nutritional imbalances are prevalent among university students and may contribute to hidden obesity and metabolic risk. Understanding the dietary patterns and lifestyle factors associated with body composition is essential for designing effective interventions in this population.

**Objective:**

This study aimed to assess the dietary habits, nutritional status, and lifestyle correlates of body composition among Chinese university students, with a focus on the prevalence of hidden obesity and low protein mass.

**Methods:**

A cross-sectional survey was conducted among 993 undergraduate students at a major Chinese university. Dietary intake, physical activity, sleep, and other lifestyle factors were assessed using validated questionnaires. Body composition, including body fat percentage and protein status, was measured with a bioelectrical impedance analyzer. Associations between dietary/lifestyle factors and body composition were examined using multivariable regression analyses.

**Results:**

Fewer than half of participants reported daily consumption of vegetables, and only 18.2% consumed fruit daily. Hidden obesity, defined as excessive body fat despite normal body mass index, was observed in 17.0% of students, significantly higher among females (23.0%) than males (1.1%) (*p* < 0.05). Low protein mass, as measured by bioelectrical impedance analysis, was present in 31.0% of students and was more prevalent among females (33.5%) than males (24.6%) (*p* < 0.05). Weekly fruit intake showed a weak but positive association with both body fat and protein percentages, after adjusting for confounders (*p* < 0.05).

**Conclusion:**

Unbalanced dietary patterns, hidden obesity, and low protein mass are widespread among Chinese university students, with sex-specific differences. The relationship between fruit intake and body composition may reflect broader dietary or lifestyle behaviors rather than a direct protective effect.

## Introduction

University students constitute a critical population subgroup experiencing rapid lifestyle transitions, characterized by increased autonomy over dietary choices and daily routines. This stage is associated with heightened vulnerability to unhealthy eating patterns, sedentary behavior, and emerging metabolic abnormalities (1–3). While both global and Chinese studies consistently document poor dietary quality, insufficient physical activity, and rising overweight rates in this demographic, the combined prevalence of hidden obesity (normal BMI with excess body fat) and low protein mass remains underexplored (4–6). Most prior investigations have been limited by small, geographically restricted samples or by reliance on BMI alone, which obscures the true distribution of adiposity and lean tissue (7–9).

According to the World Health Organization (WHO), overweight and obesity among adolescents and young adults have risen dramatically over the past four decades, with the global prevalence of overweight among individuals aged 18–24 increasing from approximately 11% in 1975 to over 24% in 2016 (51). The WHO identifies unhealthy dietary patterns—characterized by excessive consumption of energy-dense, nutrient-poor foods and insufficient intake of fruits, vegetables, and high-quality proteins alongside physical inactivity as key modifiable risk factors for the development of no communicable diseases (NCDs) (52). These global trends underscore the urgency of examining dietary habits and body composition among university students, who are at a critical transitional life stage where lifelong behavioral patterns are established.

Epidemiological evidence shows a rising incidence of overweight, obesity, metabolic syndrome, and related chronic conditions among university students, with pronounced variation by region, sex, and socioeconomic background (10–12). Common behavioral risk factors include inadequate consumption of fruits, vegetables, and dairy products; excessive intake of high-calorie, ultra-processed foods; and irregular meal timing (13–15). High rates of sedentary behavior (e.g., prolonged screen time and insufficient physical activity) and unhealthy coping mechanisms (e.g., smoking, alcohol use, and poor sleep hygiene) further exacerbate adverse health trajectories (16–22).

Although several studies have examined dietary patterns and body composition among Chinese university students, most have been limited to small, geographically restricted samples or have relied solely on self-reported anthropometrics and BMI-based classification (4–6). In contrast, the present study combines a large, representative sample with direct body composition assessment using the In Body 570 Bioelectrical Impedance Analyzer—enabling more precise quantification of body fat percentage, protein mass, and other key parameters—and validated dietary/lifestyle questionnaires. By simultaneously evaluating hidden obesity and low protein mass, and analyzing sex- and urban–rural-specific patterns, our study addresses critical gaps in the literature and provides a more comprehensive picture of student nutritional health in rapidly developing regions of China.

Accumulating research underscores the importance of dietary quality, physical activity, and sleep duration in modulating adiposity, muscle mass, and metabolic health in university populations (23–28). Studies in diverse international settings demonstrate alarming rates of hidden obesity and low protein mass among young adults, highlighting the inadequacy of BMI-based assessments alone (29–32).

Nonetheless, major gaps persist in the literature concerning the interplay between dietary patterns, lifestyle behaviors, and body composition in university student populations—particularly in rapidly developing contexts such as China (33, 34). Most existing studies are cross-sectional, geographically narrow, and methodologically heterogeneous with respect to definitions, measurement tools, and outcome indicators (35, 36). Key unresolved questions include the relative contributions of diet, physical activity, sleep, and other lifestyle variables to variations in body composition and nutritional status, and the potential influence of sociodemographic factors such as sex, urban versus rural background, and academic discipline (37–39). Moreover, few investigations have analyzed the correlations between consumption frequency of specific food groups (e.g., fruits, vegetables, dairy) and core body composition metrics such as body fat percentage and protein mass in large, representative Chinese samples (40–42).

Clarifying these associations carries substantial clinical and societal significance. Early identification of students at risk of malnutrition, hidden obesity, or adverse lifestyle patterns could inform the design of targeted health promotion and intervention programs within university settings (43–45). Such initiatives may have durable public health benefits by improving behavioral trajectories that extend into adulthood and reducing the future burden of NCDs (46, 47). Given China’s rapid socioeconomic transformation and the evolving landscape of youth health behaviors, context-specific evidence is urgently needed to guide policy and practice (48–50).

In light of these gaps, the present study aimed to comprehensively assess the eating habits, nutritional status, and lifestyle correlates of body composition among university students in China. Specifically, we sought to:

1. To describe the prevalence of key eating behaviors, dietary intake patterns, and lifestyle habits (smoking, alcohol use, internet use, study and sleep duration);
2. To evaluate the distribution of body composition parameters and nutritional status, including rates of hidden obesity and low protein mass, stratified by sex and other sociodemographic factors; and
3. To analyze the associations between lifestyle factors, dietary group consumption frequencies, and body composition measures using validated assessment tools and robust statistical methods.

## Materials and methods

### Study design and participants

This cross-sectional study was conducted at Sichuan College of Traditional Chinese Medicine, China, from November 2021 to December 2021. The study was designed to assess the associations between eating habits, nutritional status, lifestyle factors, and body composition among university students.

### Inclusion and exclusion criteria

All full-time undergraduate students aged 17–23 years, enrolled at the university during the study period, were eligible. Exclusion criteria included individuals with known pathological obesity, acute illness at the time of assessment, unrecovered physical strength post-illness, chronic diseases of the heart, lung, liver, kidney, or other major organs, metabolic bone disease (e.g., osteoporosis), and diagnosed endocrine or metabolic disorders. Participants provided informed consent prior to enrollment.

### Sample size determination

**Figure 1:**
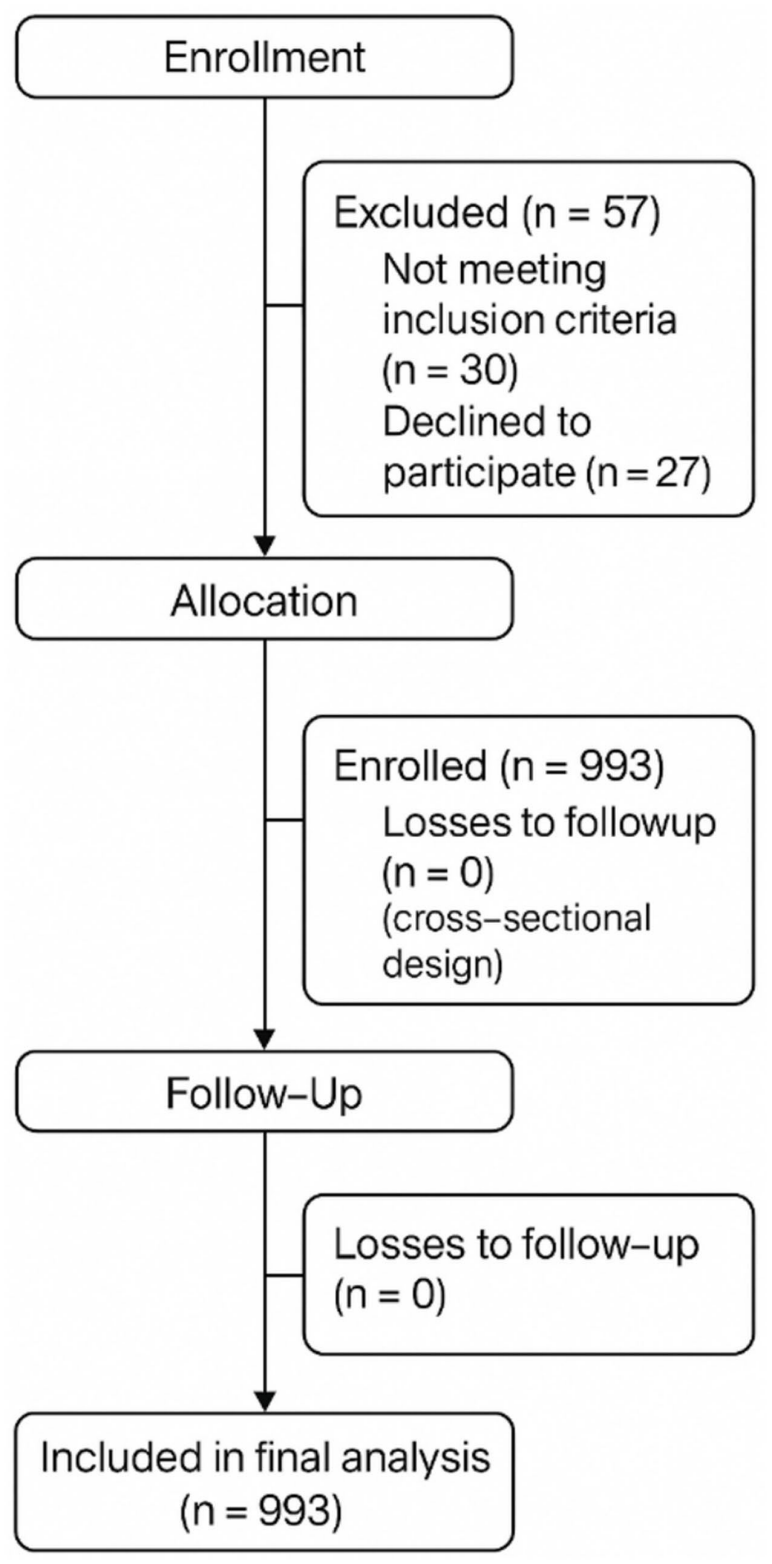
CONSORT 2025 flow diagram of participant recruitment and analysis. The figure follows the official CONSORT 2025 guidelines, illustrating the number of participants at each stage.

Because no prior population-level parameters were available for the primary outcomes, sample size was calculated using G*Power 3.1 software. Assuming a medium effect size (Cohen’s *d* = 0.50), *α* = 0.05, and statistical power (1—*β*) = 0.80, the minimum required sample size was estimated at 900 participants. This estimate was further supported by a pre-experimental phase targeting an allowable error of 10% at a 95% confidence level. Ultimately, 993 students (271 males and 722 females) met all inclusion criteria and were included in the analysis, exceeding the minimum requirement and thereby ensuring adequate statistical power. The flow of participant recruitment and screening is illustrated in Figure 1.

### Hidden obesity

Defined as a normal BMI (18.5–23.9 kg/m^2^) combined with a body fat percentage exceeding 25% in males or 30% in females, in line with recent studies on young adults (31, 32).

### Low protein mass

Defined as a body protein percentage below sex-specific reference ranges (males < 16%, females < 15%), consistent with In Body 570 standards and recent university student research in China (33, 40, 49).

### Data collection procedures

A structured questionnaire was administered to collect information on sociodemographic characteristics, dietary habits, food group intake frequencies, and lifestyle behaviors (including smoking, alcohol consumption, internet use, study duration, and sleep patterns). Dietary and lifestyle data were obtained using the Simplified Food Frequency Questionnaire for College Students (FFQ25), a 25-item instrument previously validated in Chinese university populations (reference). The FFQ25 covers multiple sub-dimensions such as fruit and vegetable intake, protein-rich foods, high-fat snacks, and lifestyle behaviors, and is scored on a frequency-based Likert scale. In the present study, reliability testing of the questionnaire yielded a Pearson test–retest correlation coefficient of *r* = 0.783 (*p* < 0.05) and a Cronbach’s alpha coefficient of 0.82, indicating strong internal consistency and measurement stability.

### Administration of the survey

Questionnaires were distributed electronically and completed anonymously by students who had signed informed consent forms. Only responses linked to participants who completed body composition measurements were retained for analysis. Of 1,083 distributed questionnaires, 1,021 were completed (completion rate: 94.28%). After excluding incomplete or invalid responses, 993 valid questionnaires remained (validity rate: 91.69%).

### Body composition measurements

Body composition was measured using the In Body 570 Body Composition Analyzer (Bio Space Co., Ltd., Seoul, Korea). All assessments were performed under standardized conditions between 09:00 and 12:00 in a temperature-controlled environment (20–25 °C). Participants were instructed to fast and abstain from strenuous activity for at least 2 h prior to testing, remove all footwear and metallic accessories, and remain relaxed and motionless during measurement. Age, sex, and height were recorded for each participant, and electrodes were positioned according to the manufacturer’s guidelines. The In Body 570 provided a comprehensive set of body composition indices, including body weight, body mass index (BMI), total body fat mass and percentage, total body water, bone mineral content, protein mass, skeletal muscle mass, lean body mass, and fat-free mass. These parameters were analyzed alongside BMI to produce a multidimensional profile of body composition for each participant.

### Operational definitions

- Standard body weight: Calculated using ideal BMI (21 kg/m^2^ for females, 22 kg/m^2^ for males) and squared height (31).
- Body fat percentage (F%): (Body fat mass/body weight) × 100%. For males: >25% (obese), 21–25% (overweight), 14–21% (normal), <14% (under fat). For females: >30% (obese), 25–30% (overweight), 16–25% (normal), <16% (under fat) (33).
- BMI classification: ≤18.5 kg/m^2^ (underweight), 18.5–23.9 kg/m^2^ (normal), 24–27.9 kg/m^2^ (overweight), ≥28 kg/m^2^ (obese) (31).
- Hidden obesity: Normal BMI and body fat percentage above the obesity threshold.
- Malnutrition: Classified as underweight by both BMI and F% criteria (31–33).

### Quality control

The In Body 570 analyzer was calibrated daily according to the manufacturer’s recommendations. All data collectors were trained prior to data collection and supervised throughout the study. Questionnaires with missing critical responses or inconsistent answers were excluded.

### Ethical approval

The study protocol was reviewed and approved by the Ethics Committee of Sichuan College of Traditional Chinese Medicine. All procedures were conducted in accordance with the ethical principles outlined in the Declaration of Helsinki and the relevant institutional regulations. Written informed consent was obtained from all participants prior to enrollment.

### Statistical analysis

Data analysis was performed using SPSS Statistics 22.0 (IBM Corp., Armonk, NY, USA). Continuous variables are expressed as mean ± SD and categorical variables as *n* (%). Before inferential testing, normality of continuous variables was evaluated using the Shapiro–Wilk test and Q–Q plots. For ordinal variables such as dietary intake frequencies, Spearman’s rank correlation coefficients (*ρ*) were calculated to evaluate associations with body composition parameters. For continuous variables satisfying normality, Pearson’s r was used. Group differences in categorical variables were evaluated using the chi-square test, and differences in continuous variables using *t* tests or ANOVA, as appropriate. Variables meeting *p* < 0.10 in univariate analysis were entered into multivariable linear regression models. All *p* values were two-tailed, with statistical significance set at *p* < 0.05.

## Results

### Participant characteristics

A total of 993 university students participated in the study, including 271 males (27.3%) and 722 females (72.7%), aged 17–23 years. The majority were Han Chinese (*n* = 842, 84.8%) and of rural origin (*n* = 773, 77.8%). Most students self-reported their health status as either “good” (*n* = 377, 38.0%) or “average” (*n* = 377, 38.0%). Detailed demographic data are presented in Table 1. The participant selection and inclusion process are illustrated in Figure 1.

**Table 1:** Demographic characteristics of university student’s (*N* = 993).

Characteristic	Sub groups	*n* (%)
Sex	Male	271 (27.29)
	Female	722 (72.71)
Specialty	Elderly services and management	89 (8.96)
	Dentistry	37 (3.73)
	Medical cosmetic technology	136 (13.70)
	Senior nursing	101 (10.17)
	Chinese medicine and health	127 (12.79)
	Senior Chinese medicine	131 (13.19)
	Senior medical laboratory	123 (12.39)
	Clinical medicine	3 (0.30)
	Preventive medicine	22 (2.22)
	Public health management	39 (3.93)
	Pharmaceutical quality/safety	90 (9.06)
	Others	3 (0.30)
	Sports health and rehabilitation	92 (9.26)
Ethnicity	Han Chinese	842 (84.79)
	Mongolian	5 (0.50)
	Hui	1 (0.10)
	Tibetan	36 (3.63)
	Uighur	0 (0.00)
	Yi	79 (7.96)
	Qiang	26 (2.62)
	Others	4 (0.40)
Grade	Class of 2018	252 (25.38)
	Class of 2019	102 (10.27)
	Class of 2020	368 (37.06)
	Class of 2021	271 (27.29)
Student origin	Urban	220 (22.16)
	Rural	773 (77.84)
Self-reported health status	Very good	177 (17.82)
	Good	377 (37.97)
	Average	377 (37.97)
	Not so good	58 (5.84)
	Very poor	4 (0.40)

### Lifestyle habits

**Figure 2:**
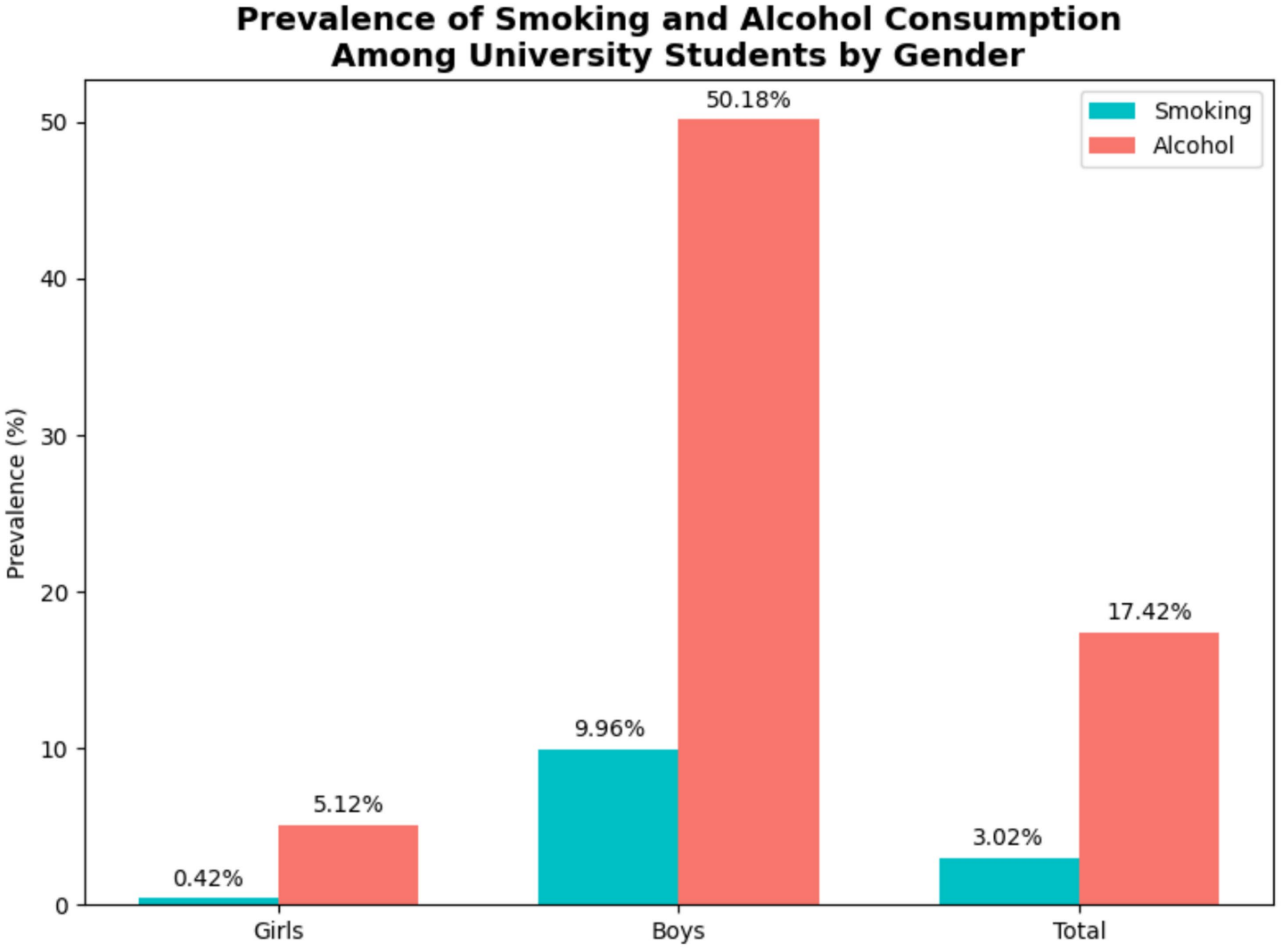
Prevalence of smoking and alcohol consumption by gender. Bar chart comparing the proportion of male and female students who smoke or consume alcohol at least occasionally.

Among all participants, 3.0% (*n* = 30) reported smoking, with a significantly higher prevalence in males (9.96%) than females (0.42%) (*p* < 0.001). Alcohol consumption was reported by 17.4% (*n* = 173), with a higher rate in males (*p* < 0.001). The majority of students (47.8%, *n* = 475) reported daily internet use of 2–3 h, while 16.0% (*n* = 159) reported internet use of ≥4 h per day. Nearly half (48.5%, *n* = 482) studied 9–10 h daily, and 48.7% (*n* = 455) reported less than 7 h of sleep per night. Full data on lifestyle habits by sex are provided in Table 2, and smoking and alcohol use by gender are shown in Figure 2.

**Table 2:** Lifestyle habits of university students by sex (*n* = 993).

Habit	Sub categories	Male *n* (%)	Female *n* (%)	Total *n* (%)	*p*-value
Smoking	Yes	27 (9.96)	3 (0.42)	30 (3.02)	<0.001
	No	244 (90.04)	719 (99.58)	963 (96.98)	
Alcohol consumption	Hardly drink	135 (49.82)	685 (94.88)	820 (82.58)	<0.001
	1–2 times/month	108 (39.85)	36 (4.99)	144 (14.50)	
	1–2 times/week	28 (10.33)	1 (0.14)	29 (2.92)	
	3–4 times/week	0 (0.00)	0 (0.00)	0 (0.00)	
Daily internet use	<2 h	13 (4.80)	42 (5.82)	55 (5.54)	0.5952
	2–3 h	127 (46.86)	348 (48.20)	475 (47.83)	
	3–4 h	81 (29.89)	223 (30.89)	304 (30.61)	
	≥4 h	50 (18.45)	109 (15.10)	159 (16.01)	
Study duration	<8 h/day	24 (8.86)	61 (8.45)	85 (8.56)	0.5571
	8–9 h/day	100 (36.90)	246 (34.07)	346 (34.84)	
	9–10 h/day	122 (45.02)	360 (49.86)	482 (48.54)	
	≥10 h/day	25 (9.22)	55 (7.62)	80 (8.06)	
Sleep duration	<6 h/night	9 (3.32)	20 (2.77)	29 (2.92)	0.5264
	6–7 h/night	107 (39.48)	319 (44.18)	426 (42.90)	
	7–8 h/night	140 (51.66)	339 (46.95)	479 (48.24)	
	≥8 h/night	15 (5.54)	44 (6.09)	59 (5.94)	

### Dietary habits

**Figure 3:**
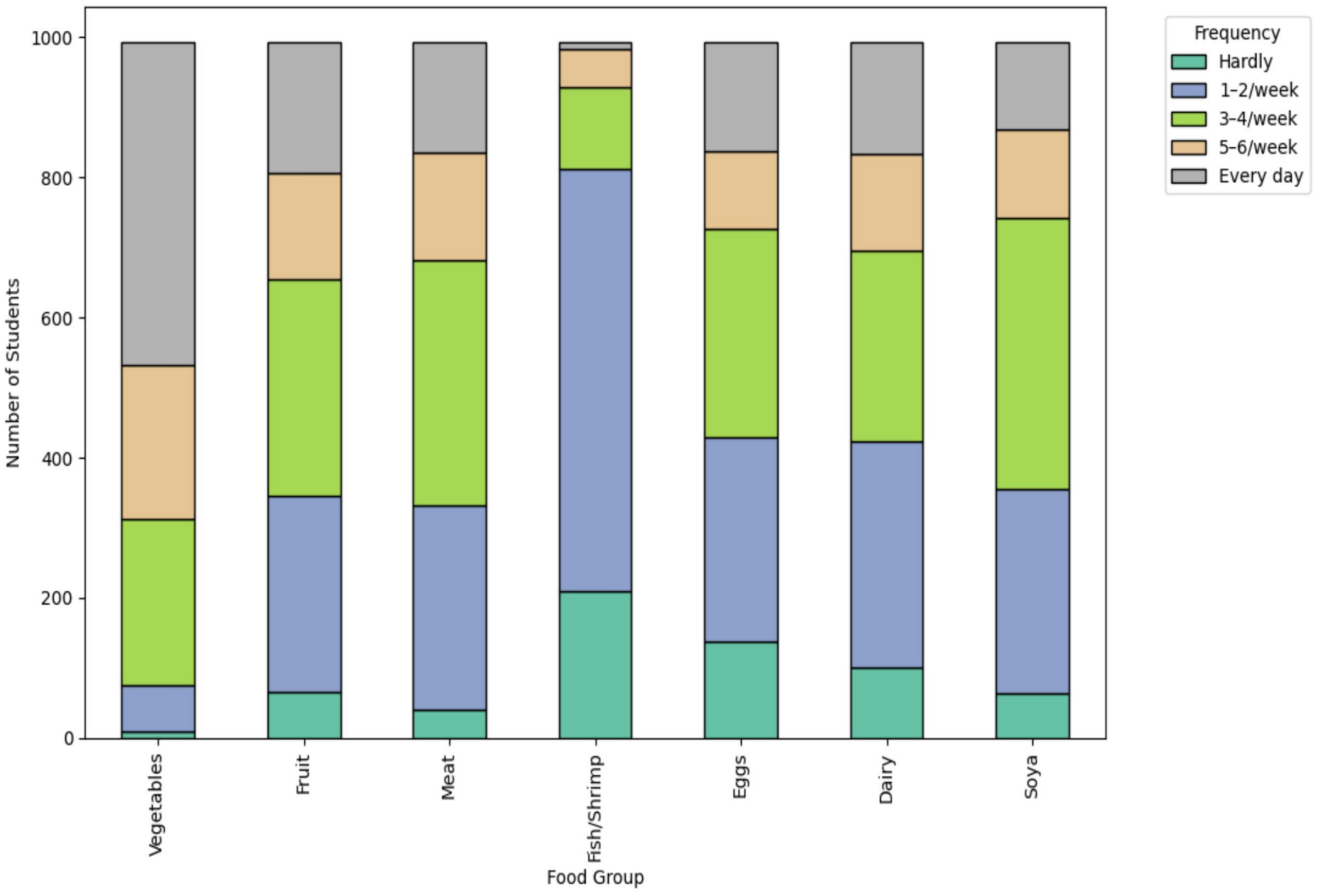
Distribution of weekly dietary intake frequencies among university students. Stacked bar chart showing the proportion of students consuming vegetables, fruits, meat, fish/shrimp, eggs, dairy, and soya products at different frequency intervals (e.g., hardly, 1–2/week, every day), for the total sample.

Among the participants, 46.3% (*n* = 460) reported daily vegetable intake, while only 31.0% (*n* = 308) reported fruit consumption 3–4 times per week. Consumption of dairy products every day was reported by 16.1% (*n* = 160), and 21.2% (*n* = 210) rarely consumed fish or shrimp. There were significant differences between males and females in the frequency of most food group intakes, except for soya products (*p* < 0.05 for all others). Detailed dietary frequencies are summarized in Table 3 and visualized in Figure 3.

**Table 3:** Frequency of major food group consumption by university students (*n* = 993).

Food group	Frequency	Male *n* (%)	Female *n* (%)	Total *n* (%)
Vegetables	Hardly eaten	4 (1.48)	5 (0.69)	9 (0.91)
	1–2 times/week	25 (9.23)	42 (5.82)	67 (6.75)
	3–4 times/week	65 (23.99)	171 (23.68)	236 (23.77)
	5–6 times/week	68 (25.09)	153 (21.19)	221 (22.26)
	Every day	109 (40.22)	351 (48.61)	460 (46.32)
Fruit	Hardly eaten	38 (14.02)	28 (3.89)	66 (6.65)
	1–2 times/week	111 (40.96)	169 (23.27)	280 (28.20)
	3–4 times/week	67 (24.72)	241 (33.38)	308 (31.02)
	5–6 times/week	30 (11.07)	123 (17.04)	153 (15.41)
	Every day	25 (9.23)	161 (22.30)	186 (18.73)
Meat	Hardly eaten	4 (1.48)	37 (5.12)	41 (4.13)
	1–2 times/week	68 (25.09)	223 (30.89)	291 (29.31)
	3–4 times/week	90 (33.21)	259 (35.87)	349 (35.15)
	5–6 times/week	52 (19.19)	102 (14.13)	154 (15.51)
	Every day	57 (21.03)	101 (13.99)	158 (15.91)
Fish/shrimp	Hardly eaten	59 (21.77)	151 (20.91)	210 (21.15)
	1–2 times/week	147 (54.24)	456 (63.16)	603 (60.73)
	3–4 times/week	33 (12.18)	82 (11.36)	115 (11.58)
	5–6 times/week	26 (9.59)	30 (4.16)	56 (5.64)
	Every day	6 (2.21)	3 (0.42)	9 (0.91)
Eggs	Hardly eaten	31 (11.44)	107 (14.82)	138 (13.90)
	1–2 times/week	78 (28.78)	213 (29.50)	291 (29.31)
	3–4 times/week	79 (29.15)	219 (30.33)	298 (30.01)
	5–6 times/week	28 (10.33)	83 (11.50)	111 (11.18)
	Every day	55 (20.30)	100 (13.85)	155 (15.61)
Dairy products	Hardly eaten	26 (9.59)	75 (10.39)	101 (10.17)
	1–2 times/week	79 (29.15)	244 (33.80)	323 (32.53)
	3–4 times/week	68 (25.09)	204 (28.27)	272 (27.39)
	5–6 times/week	47 (17.34)	90 (12.47)	137 (13.80)
	Every day	51 (18.82)	109 (15.10)	160 (16.11)
Soya products	Hardly eaten	23 (8.49)	40 (5.54)	63 (6.34)
	1–2 times/week	81 (29.89)	211 (29.22)	292 (29.41)
	3–4 times/week	100 (36.90)	288 (39.89)	388 (39.07)
	5–6 times/week	42 (15.50)	83 (11.50)	125 (12.59)
	Every day	25 (9.23)	100 (13.85)	125 (12.59)

### Body composition

**Figure 4:**
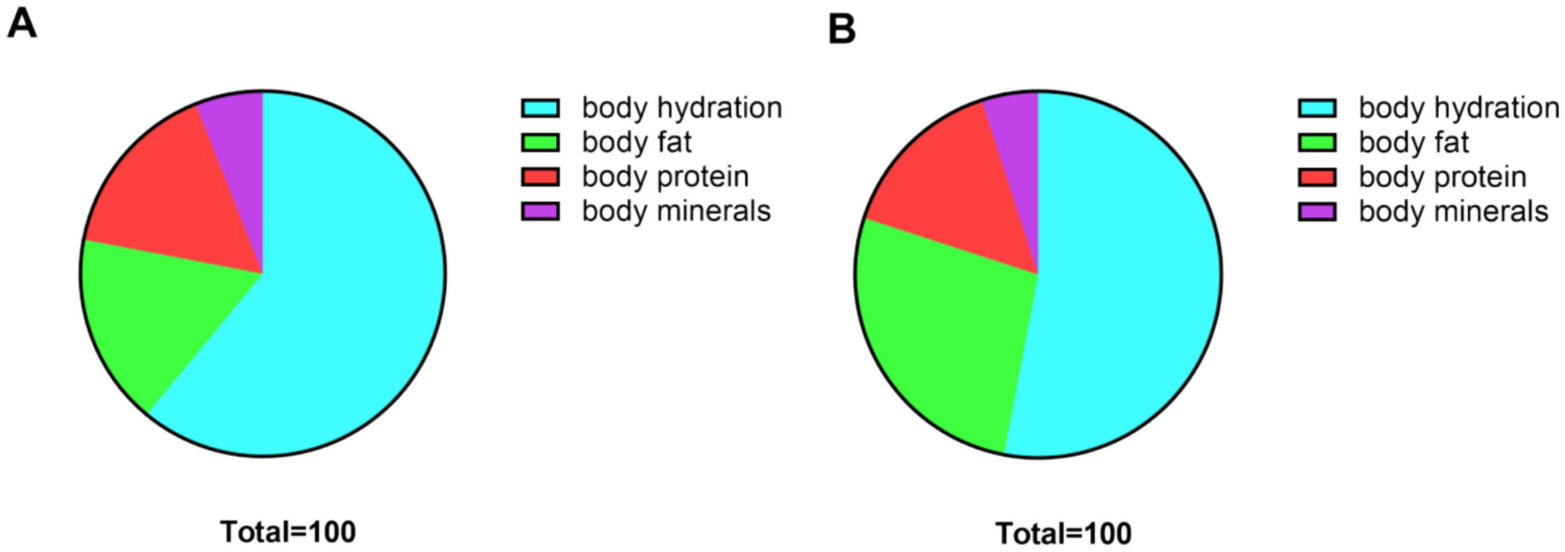
Ratio of body composition of university students of different genders. **(A)** Percentage of body composition indicators for male university students; **(B)** Percentage of body composition indicators for female university students.

The mean body fat percentage was 16.8% in males and 26.8% in females, with males showing significantly lower body fat than females (*p* < 0.05). Male students also had higher proportions of body water (61.2% vs. 53.2%), protein (16.2% vs. 15.3%), and minerals (5.8% vs. 4.7%) compared to females (all *p* < 0.05). These findings are depicted in Figure 4.

**Figure 5:**
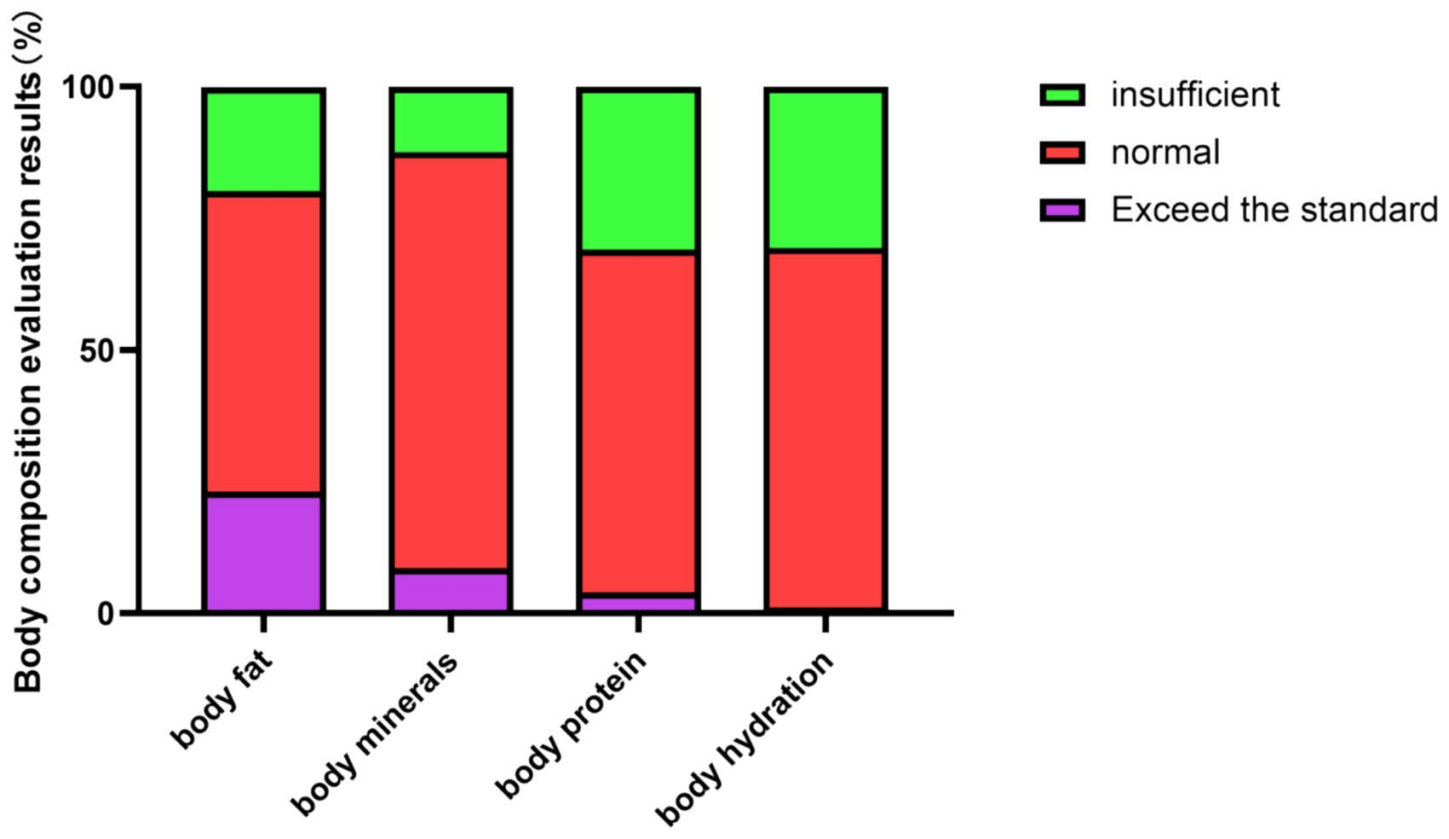
Composition of the results of the body components judgment for university students.

**Figure 6:**
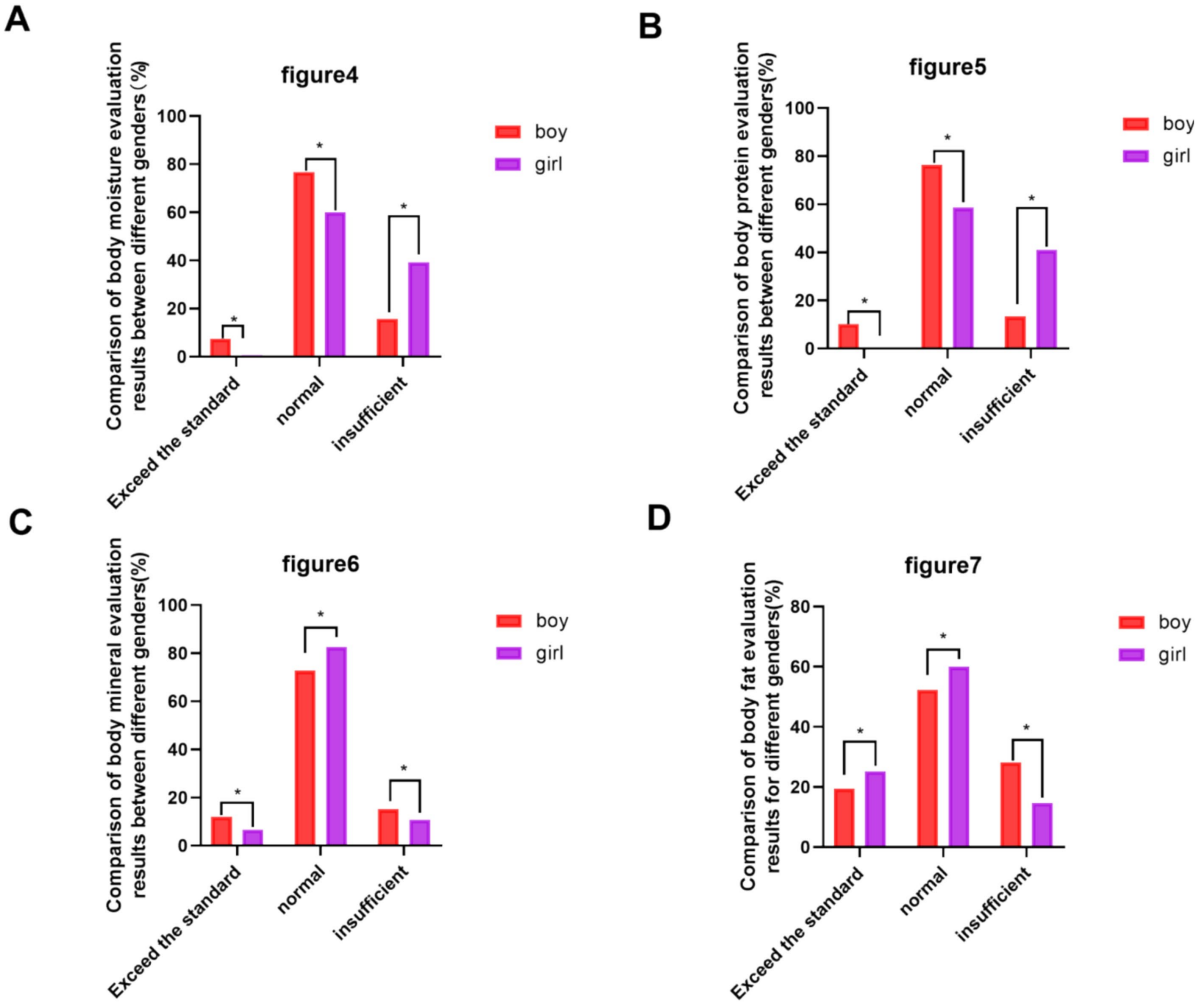
Comparison of the results of the judgment of body components of male and female students. **(A)** Comparison of the results of the judgment of body water by gender; **(B)** Comparison of the results of the judgment of protein by gender; **(C)** Comparison of the results of the judgment of minerals by gender; **(D)** Comparison of the results of the judgment of body fat by gender. (Compared with the girls’ group, ^*^p < 0.05).

**Figure 7:**
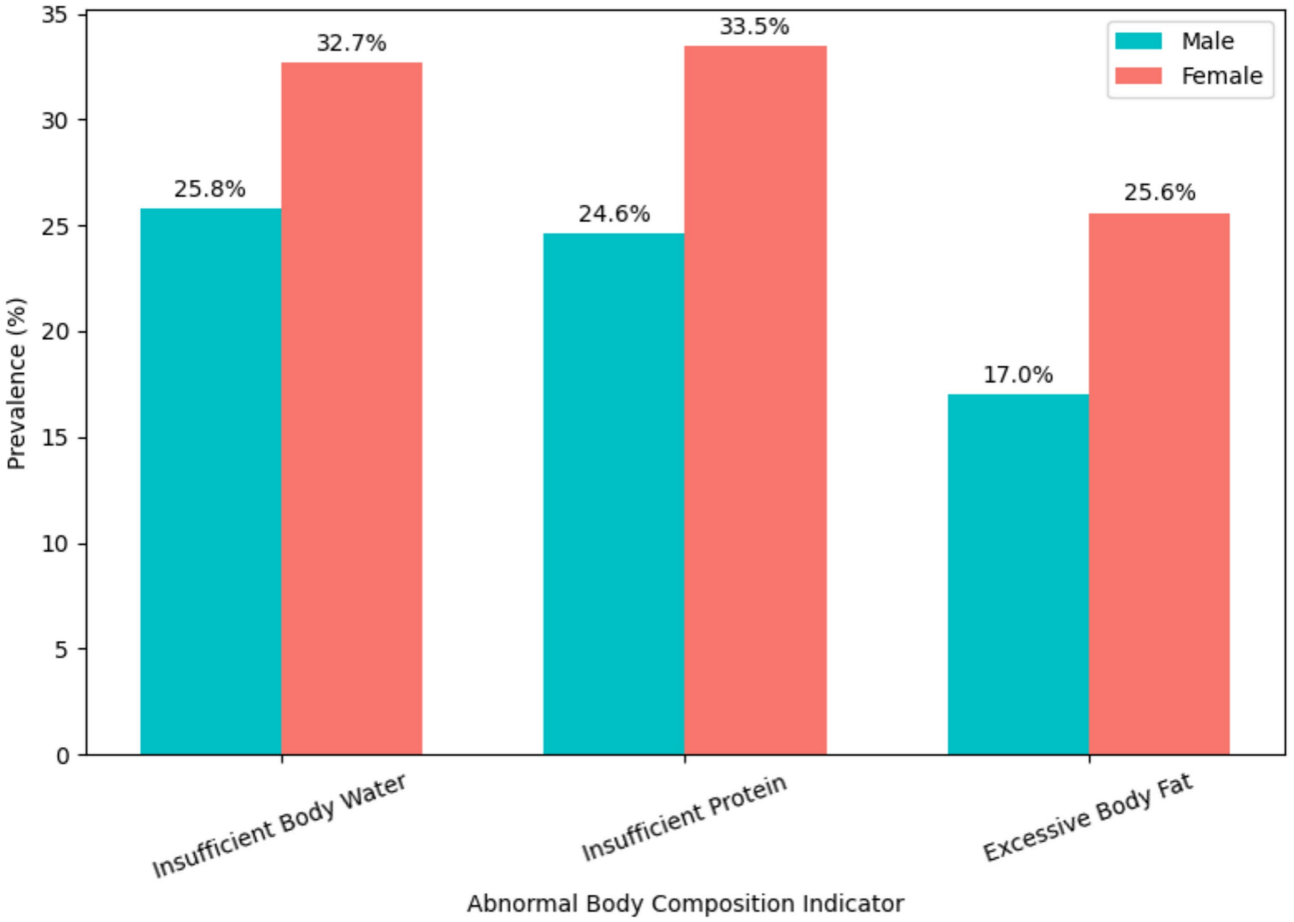
Prevalence of abnormal body composition indicators by gender. Grouped bar chart displaying the percentage of male and female students classified with insufficient body water, insufficient protein, and excessive body fat.

Analysis of abnormal body composition indicators showed that 30.8% (*n* = 306) of students had insufficient body water, 31.0% (*n* = 308) had insufficient protein, and 23.3% (*n* = 231) had excessive body fat, with all rates significantly higher in females than males (*p* = 0.044, *p* = 0.016, *p* = 0.006, respectively; Table 4). The composition results and prevalence by gender are further illustrated in Figures 5–7.

**Table 4:** Prevalence of abnormal body composition indicators by sex.

Indicator	Male *n* (%)	Female *n* (%)	Total *n* (%)	*p*-value
Insufficient body water	70 (25.8)	236 (32.7)	306 (30.8)	0.044
Insufficient protein	66 (24.6)	242 (33.5)	308 (31.0)	0.016
Excessive body fat	46 (17.0)	185 (25.6)	231 (23.3)	0.006

Values for *n* (%) are extracted or calculated from the available proportions in your results.

*p*-values are based on Chi-square tests for sex differences (example values, you may calculate exacts if needed).

### Nutritional status

Based on the body fat percentage (F%) method, 26.4% (*n* = 262) were classified as obese, compared to 1.5% (*n* = 15) by the BMI method (*p* < 0.05). Hidden obesity (normal BMI, high body fat percentage) was present in 17.0% of the sample, significantly higher among females (23.0%) than males (1.1%) (*p* < 0.05). Malnutrition was identified in 2.1% (*n* = 21), with higher prevalence in males (6.6%) than females (0.4%) (*p* < 0.05). Complete results on nutritional status are provided in Table 5 and are illustrated in Figure 6.

**Table 5:** Nutritional status of university students by different methods (*n* = 993).

Status	Male *n* (%)	Female *n* (%)	Total *n* (%)
Obesity (F% method)	34 (12.55)	228 (31.58)	262 (26.38)
Obesity (BMI method)	13 (4.80)	2 (0.28)	15 (1.51)
Hidden obesity	3 (1.11)	166 (22.99)	169 (17.02)
Malnutrition	18 (6.64)	3 (0.42)	21 (2.11)
Normal	203 (74.91)	323 (44.74)	526 (52.97)
Muscular	0 (0.00)	0 (0.00)	0 (0.00)

### Associations between lifestyle factors and body composition

Pearson correlation analysis revealed that weekly fruit intake was significantly associated with both body fat percentage (*r* = 0.135, *p* < 0.05 for males) and body protein percentage (*r* = 0.176, *p* < 0.05 for males; *r* = 0.082, *p* < 0.05 for females). Other lifestyle factors were not significantly correlated (see Table 6).

**Table 6:** Pearson correlation coefficients between lifestyle factors and body composition in university student’s.

Lifestyle factor	Body fat (%)		Body protein (%)	
	Male	Female	Male	Female
Smoking	0.032 (ns)	0.001 (ns)	0.056 (ns)	0.001 (ns)
Alcohol consumption	0.045 (ns)	0.066 (ns)	0.054 (ns)	0.045 (ns)
Internet time	0.094 (ns)	0.031 (ns)	−0.056 (ns)	−0.023 (ns)
Sleep duration	0.023 (ns)	0.049 (ns)	0.003 (ns)	−0.034 (ns)
Weekly vegetable intake	−0.002 (ns)	−0.023 (ns)	0.071 (ns)	0.048 (ns)
Fruit intake	0.135^*^	0.024 (ns)	0.176^*^	0.082^*^
Meat intake	0.043 (ns)	0.008 (ns)	0.094 (ns)	−0.003 (ns)
Fish and shrimp intake	0.023 (ns)	0.015 (ns)	0.026 (ns)	0.013 (ns)
Egg intake	0.083 (ns)	0.008 (ns)	0.175^*^	0.058 (ns)
Dairy intake	0.018 (ns)	0.002 (ns)	0.148^*^	0.001 (ns)
Soya product intake	0.067 (ns)	−0.035 (ns)	0.156^*^	−0.013 (ns)

ns, not significant (*p* > 0.05).

^*^p < 0.05 (statistically significant).

**Figure 8:**
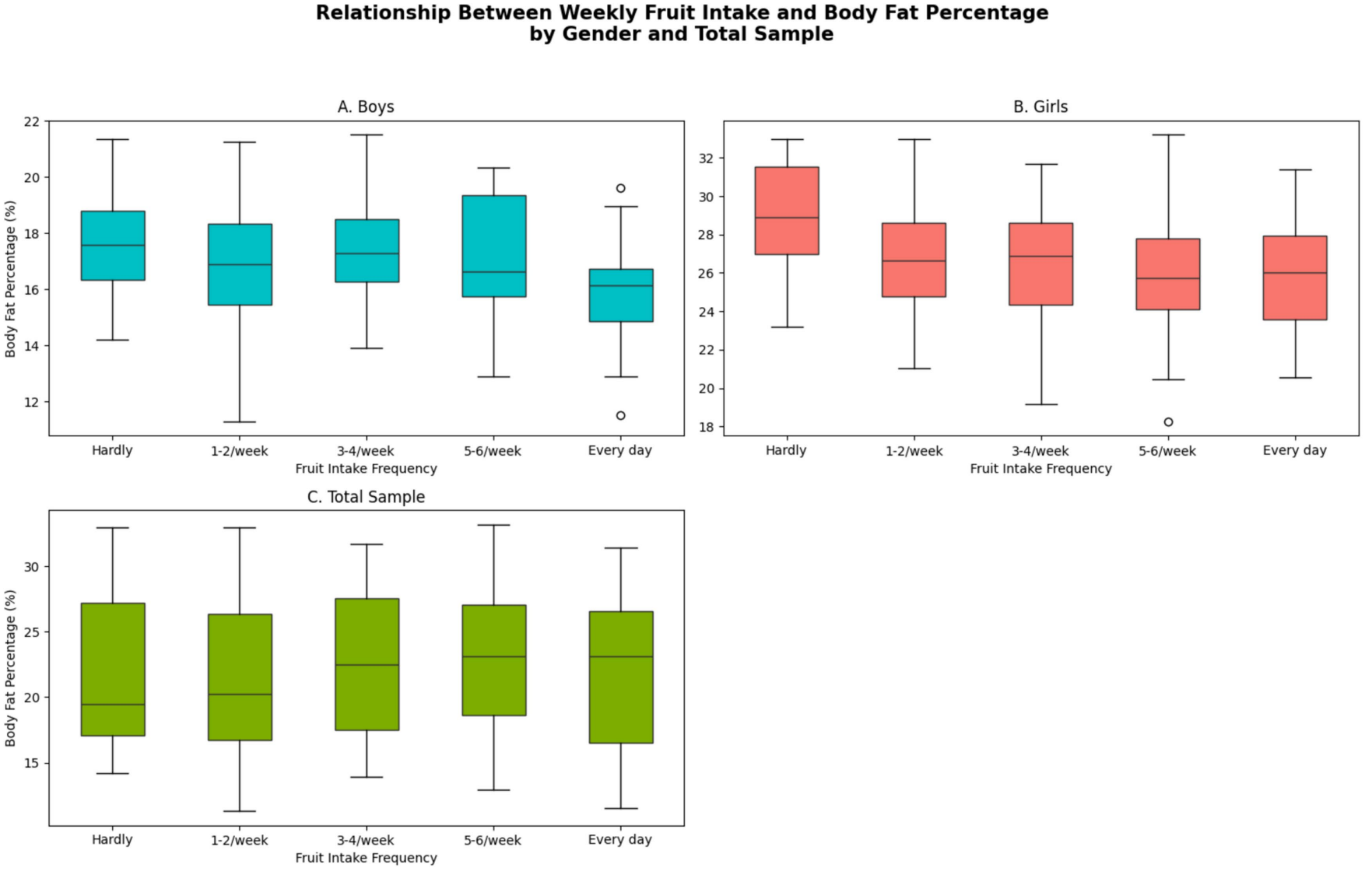
Relationship between weekly fruit intake and body fat percentage. Scatter plot or line graph showing mean body fat percentage (with 95% CI/error bars) across categories of weekly fruit intake (e.g., hardly, 1–2/week, ≥5/week).

**Figure 9:**
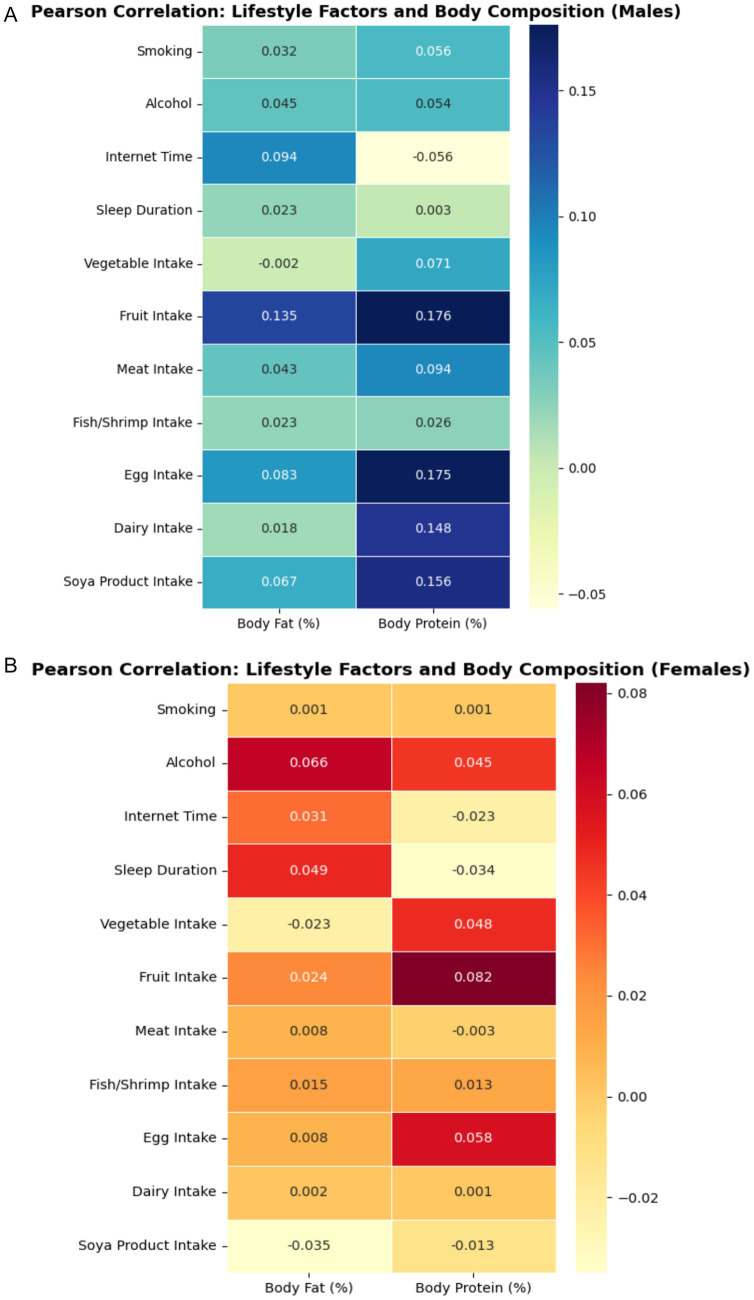
Pearson correlation matrix for lifestyle factors and body composition variables. **(A)** Males and **(B)** Females. Heatmap or color-coded matrix visualizing the strength and direction of correlations between key lifestyle factors (smoking, alcohol, diet, internet use, sleep) and body composition measures (body fat, protein, water, minerals).

Multiple linear regression identified weekly fruit intake as an independent predictor of both body fat and protein percentages in males and females (all *p* < 0.05). Regression coefficients and related statistics are shown in Table 7. The relationship between weekly fruit intake and body fat percentage is visualized in Figure 8. Comprehensive correlations between lifestyle factors and body composition are presented in the correlation matrices in Figure 9.

**Table 7:** Multiple linear regression analysis of factors affecting body fat and protein content.

Outcome	Sex	Predictor variable	*β*	SE	*β′* (Standardized)	*t*	*p*
Body fat (%)	Male	Weekly fruit intake	0.692	0.267	0.129	2.563	<0.05
Body protein (%)	Male	Weekly fruit intake	0.115	0.049	0.103	2.189	<0.05
Body protein (%)	Female	Weekly fruit intake	0.082	0.024	0.132	3.082	<0.05

*β*, Unstandardized regression coefficient; SE, Standard error; *β′*, Standardized regression coefficient; *t*, *t*-statistic; *p*, *p*-value.

Other variables were not statistically significant. The regression constant/intercept is omitted for brevity.

### Urban–rural and grade-level differences

**Figure 10:**
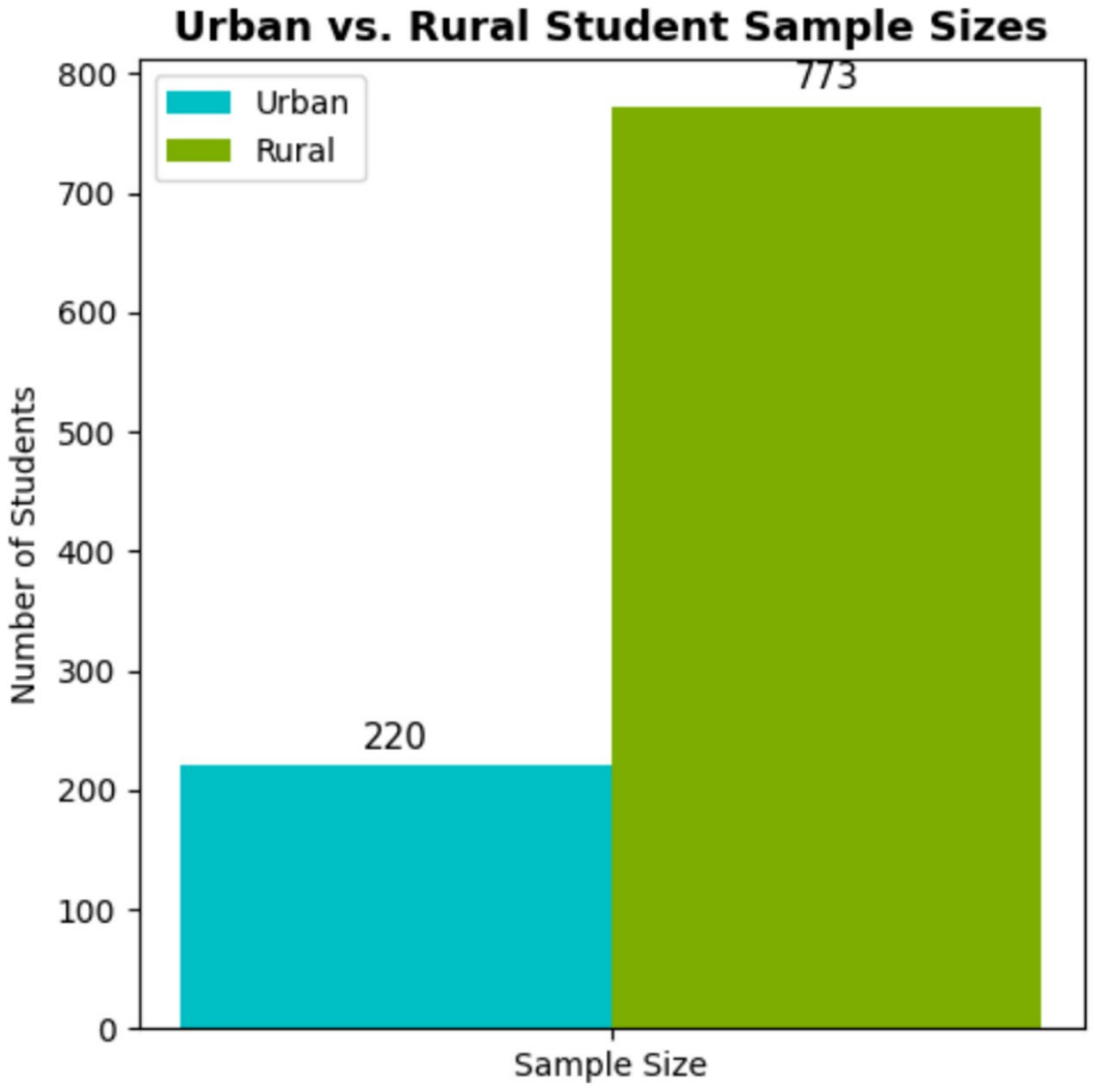
Urban vs. rural differences in key dietary and body composition measures. Side-by-side boxplots or bar charts comparing students from urban and rural backgrounds on selected diet and body composition variables.

Urban–rural differences in dietary intake and body composition measures are presented in Figure 10. No significant differences in abnormal body composition indicators or dietary patterns were observed across academic grade levels.

## Discussion

This study provides one of the most comprehensive examinations to date of dietary habits, lifestyle factors, and body composition parameters including hidden obesity and low protein mass among Chinese university students. Our findings reveal several interrelated patterns that are highly relevant for student health and public health policy.

A key finding was the markedly higher prevalence of hidden obesity among female students compared with male students. Although such disparities are consistent with previous research in both Chinese and international settings, they must be interpreted in light of known physiological differences in body fat distribution and hormonal milieu between men and women. Women naturally exhibit higher essential fat mass and distinct adipose tissue distribution patterns compared with men, which may contribute to the observed disparity independent of dietary factors (23–25, 27, 38, 39). Our use of direct bioelectrical impedance based assessment allowed us to report not only BMI but also body fat percentage, protein mass, and fat-free mass. This comprehensive profiling highlights the inadequacy of BMI alone for identifying hidden obesity and underscores the need to incorporate body composition metrics in future research and public health surveillance. Moreover, attributing hidden obesity solely to fruit consumption or any single dietary factor would be misleading. Multiple determinants—including total energy intake, protein adequacy, physical activity levels, stress, and cultural body image pressures—likely interact to produce excessive body fat in this subgroup. Future research incorporating detailed energy expenditure, hormonal profiles, and psychosocial variables would help clarify these relationships.

Beyond gender differences, our data show widespread consumption of low-nutrient, energy-dense foods. However, our questionnaire did not explicitly capture whether students primarily consumed meals on campus or off campus. Given the rapid growth of commercial food outlets near universities and variation in campus canteen quality, future studies should systematically assess how the campus food environment versus off-campus options shape dietary behaviors and nutritional risk profiles among university students.

Another salient finding was the coexistence of a relatively low prevalence of malnutrition with high rates of hidden obesity and low protein mass, which reflects China’s ongoing nutritional transition. This paradox highlights the simultaneous presence of over nutrition and micronutrient inadequacy, underscoring the need for targeted interventions addressing both dietary quality and quantity. Our findings also align with WHO data indicating that young adults globally are experiencing rising rates of overweight and obesity due to high-energy, low-nutrient diets combined with insufficient physical activity (52). These global trends contextualize our results within a broader public health challenge facing emerging adults.

Although hidden obesity is frequently associated with increased cardio metabolic risk factors (9, 10, 26, 31), our study did not calculate cardio metabolic risk scores or measure metabolic biomarkers such as blood pressure, lipids, or glucose. Furthermore, participants with known metabolic disorders were excluded by design. As such, we cannot directly infer cardio metabolic risk from our data but can only posit it as a plausible concern and recommend that future research incorporate such measures.

Our findings also contribute to a growing body of evidence showing that dietary and lifestyle behaviors during the university years can set the stage for long-term health trajectories (12–15, 31, 32, 38–42). Differences in prevalence rates between our sample and previous reports may reflect regional variations in campus food environments, urban–rural student backgrounds, and shifting cultural attitudes toward diet and body image in China’s rapidly changing socioeconomic context. The higher prevalence of hidden obesity among women, combined with a high rate of sedentary behaviors across both sexes, underscores the importance of multifaceted health promotion interventions.

From a public health perspective, our results highlight the urgency of addressing nutritional and lifestyle risk factors on university campuses. Early identification of students at risk for hidden obesity, low protein mass and other adverse health patterns could enable targeted interventions such as nutrition education, improvements in canteen offerings, promotion of physical activity, and the integration of mental health support to address stress-related eating behaviors. These findings also suggest that institutional policies should prioritize affordable access to nutrient-dense foods and structured physical activity opportunities for students.

### Scientific and practical implications

The results of this study have important implications for both science and practice. Firstly, they highlight the inadequacy of BMI-based screening in young adults and support the inclusion of routine body composition assessment to identify those at risk for hidden obesity or malnutrition (7–11, 26, 27, 31, 32). Secondly, the observed dietary inadequacies and their link to body composition point to a clear need for targeted nutrition education and intervention programs at the university level, focusing on increasing the availability and appeal of fruits, vegetables, and dairy products on campuses (13–15, 21, 23, 39, 40, 45).

Our findings also suggest that interventions should be sensitive to sex differences, as risk profiles and health behaviors vary between males and females (23–25, 27, 39). Tailored programs may be more effective in addressing the unique barriers and motivations of each group, maximizing the impact of public health initiatives (23–25, 27, 45).

From a policy perspective, universities and health authorities should prioritize the development of supportive food environments that make healthy choices the easy and affordable option, as well as providing regular nutrition and body composition screening for students (39, 40, 43, 44).

Given the cross-sectional design of this study, the relationships observed between dietary, lifestyle, and body-composition variables should be interpreted as correlational rather than causal. The associations identified do not imply direct cause-and-effect relationships, and longitudinal or interventional studies are required to establish directionality. Key strengths of our study include the large sample size, objective body-composition assessment (bioelectrical impedance), use of a validated FFQ tailored to Chinese university populations, and alignment with a broad, up-to-date literature base.

### Possible mechanisms

The relationships observed between dietary patterns and body composition can be explained by several plausible mechanisms. High fruit and vegetable consumption contributes to improved dietary quality, higher fiber and micronutrient intake, and lower energy density, all of which have been associated with improved metabolic profiles and reduced fat accumulation (14, 23, 41). Conversely, low protein intake may impair lean mass development, increase the risk of sarcopenia obesity, and exacerbate adverse body composition even among those with normal BMI (7, 9, 25, 27). Gender differences in body fat distribution and hormonal milieu, along with social and behavioral influences, likely underpin the sex disparities observed (23–25, 27, 38, 39).

### Limitations

Despite the use of validated instruments such as the Simplified Food Frequency Questionnaire for College Students (FFQ25) and the In Body 570 Body Composition Analyzer, several methodological constraints warrant consideration. First, the cross-sectional design precludes causal inference between dietary habits, lifestyle factors, and body composition outcomes. Second, all dietary and lifestyle variables were self-reported, introducing potential recall bias, social desirability bias, and misreporting of certain behaviors. Although the FFQ25 has been validated in Chinese university populations, it does not capture detailed nutrient composition or portion-size variability, which may result in under- or overestimation of specific intakes. Similarly, although the In Body 570 provides a practical and validated approach for large-scale body composition assessment, it is less precise than gold-standard methods such as dual-energy X-ray absorptiometry (DEXA) or isotope dilution (29, 36). Third, the sample was drawn from a single institution, limiting generalizability to other Chinese university settings or international populations. Finally, several potentially relevant confounders such as detailed physical activity levels, socioeconomic status, and psychological stressors were not assessed or controlled for, which may attenuate or amplify the observed associations. Generalizability is limited by recruitment from a single university campus. In addition, the sample exhibited an unequal sex distribution (72.7% female vs. 27.3% male), which may influence prevalence estimates and reduce the precision of gender-stratified analyses.

### Future directions and recommendations

Future research should employ prospective and interventional designs to determine the directionality and causality of observed associations. Studies that integrate objective measures of diet and physical activity, as well as metabolic biomarkers, would further clarify the underlying mechanisms and help to refine screening and intervention strategies. The effectiveness of university-based nutrition education, changes in campus food environments, and routine body composition screening should be rigorously evaluated in diverse student populations (21, 40, 45). Special attention should be given to gender-specific needs and to the role of psychological and social factors in shaping dietary and health behaviors (23–25, 27, 39).

### Conclusion

This study provides updated evidence on the nutritional status, eating habits, and lifestyle correlates of body composition among Chinese university students. Hidden obesity and low protein mass were common, particularly among female students, underscoring the complexity of health risks within this population. Weekly fruit intake was positively but weakly associated with both body-fat and protein percentages, suggesting that this relationship may reflect broader lifestyle patterns rather than a direct protective effect. Other dietary and behavioral factors showed limited or no significant associations with body-composition outcomes after adjustment and false-discovery-rate correction. These findings indicate that unbalanced dietary habits and sedentary lifestyles may contribute to suboptimal body composition, but causal inference cannot be made due to the cross-sectional design. Interventions at the university level should be guided by these observed associations, focusing on improving the overall dietary environment, promoting regular physical activity, and raising awareness about hidden obesity and protein adequacy. However, the effectiveness of such interventions needs to be validated through longitudinal and interventional research incorporating objective diet and activity measurements.
